# Out-of-Plane Continuous Electrostatic Micro-Power Generators

**DOI:** 10.3390/s17040877

**Published:** 2017-04-16

**Authors:** M. A. E. Mahmoud, E. M. Abdel-Rahman, R. R. Mansour, E. F. El-Saadany

**Affiliations:** 1 Department of Electrical and Computer Engineering, Ain Shams University, 1156 Cairo, Egypt; 2 Department of Systems Design Engineering, University of Waterloo, Waterloo, N2L 3G1 ON, Canada; eihab@uwaterloo.ca; 3 Department of Electrical and Computer Engineering, University of Waterloo, Waterloo, N2L 3G1 ON, Canada; rrmansour@uwaterloo.ca (R.R.M.); ehab@uwaterloo.ca (E.F.E.-S.)

**Keywords:** micro-power generators, electrostatic, out-of-plane, wideband

## Abstract

This paper presents an out-of-plane electrostatic micro-power generator (MPG). Electret-based continuous MPGs with different gaps and masses are fabricated to demonstrate the merits of this topology. Experimental results of the MPG demonstrate output power of 1 mW for a base acceleration amplitude and frequency of 0.08 g and 86 Hz. The MPGs also demonstrate a wideband harvesting bandwidth reaching up to 9 Hz. A free-flight and an impact mode model of electrostatic MPGs are also derived and validated by comparison to experimental results.

## 1. Introduction

Advances in low-power integrated circuits have led to the realization of embedded sensors with low power requirements. Combining these sensors with sustainable energy sources will allow the deployment of autonomous sensor arrays. Energy harvesting of ambient vibrations is a promising direction to satisfy this need. Vibrations are widely available in domestic and industrial environments. Vibration-based micro-power generators (MPGs) have a demonstrated ability to generate electric power in the micro- to milli-watt range [1].

In addition to the amount of generated power, a low cost implementation is a requirement for using MPGs in autonomous sensors. Among vibration harvesting techniques, electrostatic MPGs have the lowest implementation cost since no specialty materials are needed, such as magnetic materials used in electromagnetic MPGs and ceramic materials used in piezoelectric MPGs. On the other hand, the need for a charge source and switching losses adds limitations to electrostatic MPGs.

Continuous mode electrostatic MPGs are designed to eliminate the need for switched circuits. Many of them use electrets, quasi-permanently-charged dielectrics, to induce charges on the capacitor plates and eliminate the need for an initial charge source. Due to these advantages, significant efforts have been devoted to developing electret-based electrostatic MPGs. Boland et al. [2] miniaturized an electret-based generator proposed by Tada [3]. The fabricated MPG had a rotor diameter of 8 mm and output power of 25 μW. Sterken et al. [4] proposed and fabricated an electret-based electrostatic MPG using in-plane comb-finger variable capacitors and a predicted output power of 50 μW.

Tsutsumino and co-workers [5,6] developed the transducer (electric subsystem) of an electrostatic MPG using electret-based parallel-plate capacitors. A shaker moved an electrode of the variable capacitor parallel to a fixed electrode (in-plane). Using 10×20 mm${}^{2}$ electrodes, they realized a maximum of 278 μW output power. They found that dividing their in-plane variable capacitor into two out-of-phase capacitors improves the power extraction capacity of the MPG and transforms the electric damping force from Coulomb to viscous-like [6,7].

Edamoto et al. [8] constructed a fully-functioning MPG by combining this transducer with a mechanical oscillator. The movable electrodes were attached to an inertial mass supported by soft parylene springs to create a low-frequency resonator [9]. They also used two electret layers to induce levitation forces in the movable electrodes, thereby protecting against pull-in [10]. The simulated output power was 12.5 μW, but it dropped in experimental measurements to 0.28 μW due to misalignment between the electrodes [11].

Bartsch et al. [12] fabricated two MPG prototypes based on Sterken et al.’s topology: meso-sized (59×86 mm${}^{2}$) and micro-sized (3×6 mm${}^{2})$ with output powers of 0.36 μW and 1.4 nW, respectively [13]. Hoffmann et al. [14] realized another implementation of Sterken et al.’s topology that had a packaged volume of 0.2 cm${}^{3}$, and was able to produce 3.5 μW at 13 g acceleration amplitude.

Mahmoud et al. [15] proposed another implementation of Sterken et al.’s topology using parallel-plate capacitors. Their analysis of the new topology showed output power improvement to 89 μW for the same MPG size and operation conditions as [4]. Tao et al. [16] realized a similar electrostatic harvester and realized 0.1 μW output power at an excitation of 0.2 g.

Tao et al. [17] developed a micro-sized (3 mm radius) parallel-plate electrostatic MPG that harvests in-plane and out-of-plane vibrations. They found that given similar input power levels, the out-of-plane mode was more effective with output power of 4.8 nW compared to 0.67 nW and 1/2 nW for two in-plane modes. They increased the output power of the out-of-plane mode to 950 nW [18] by using two electret layers instead of one and increasing the excitation level from 0.05 g to 0.48 g. Further, they found that adding a stopper layer of PDMS on top of one of the electret layers expands the harvesting bandwidth to 3.7 Hz in frequency up-sweep and 2.8 Hz in frequency down-sweep.

In most reported literature, in-plane configurations are used to implement the capacitive transducer due to the low damping of these motions. However, such implementations require small vertical feature sizes, as well as high aspect supports to protect against out-of-plane disturbances.

In this paper, we compare in-plane and out-of-plane transducers and report on an out-of-plane electret-based electrostatic MPG. Test results demonstrate 1 mW of output power at less than 0.08 g excitation acceleration. Moreover, the prototype exhibits a wideband operation that reaches 9 Hz. We also report on two models for free-flight and impact MPGs. The simulation results obtained from these models show good agreement with the experimental results.

In Section 2, in-plane and out-of-plane transducers are compared, and the model for continuous electrostatic transducers in free-flight is derived. In Section 3, the derived model is validated by comparing its results to experimental results. Section 4 demonstrates implantation of an electret-based out-of-plane MPG. A model for impact mode electrostatic MPGs is derived and validated using the experimental results in Section 5. An improved version of the MPG is presented in Section 6. Finally, conclusions are drawn in Section 7.

## 2. Basic MPG Model

**Figure 1:**
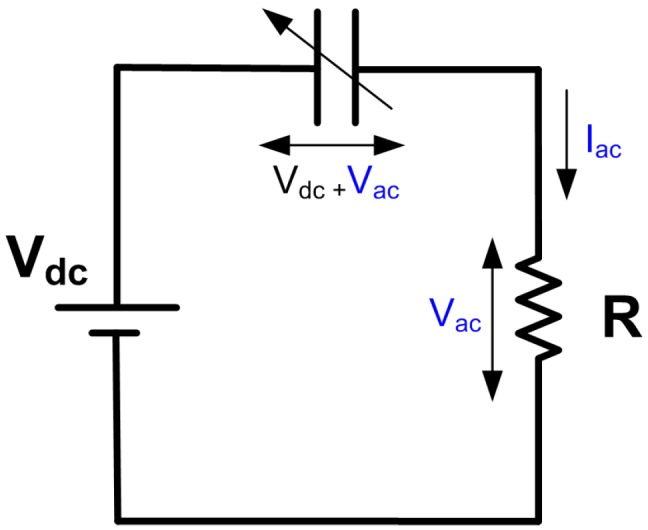
Circuit diagram for electrostatic energy harvesting.

Electrostatic energy harvesters (Figure 1) consist of a variable capacitor $C_{v}$, a DC voltage source $V_{dc}$ and a load resistance *R*. When vibrations are allowed to change the relative positions of the capacitor plates, capacitance varies over time $C_{v}\left(t\right)$, AC voltage $V_{ac}\left(t\right)$ develops across the capacitor and a current $I_{ac}\left(t\right)$ is delivered to the load. The electrical model of this electrostatic transducer can be derived by applying Kirchhoff’s voltage law to the circuit shown in Figure 1:

(1) $$V_{dc}=\frac{q}{C_{v}}+\dot{q}R$$

where q(t) is the charge stored in the capacitor and $\dot{q}=I_{ac}$. Rearranging, we obtain:

(2) $$\dot{q}=\frac{V_{dc}}{R}-\frac{q}{RC_{v}}$$

which suggests that the current delivered to the resistor depends on $C_{v}$.

**Figure 2:**
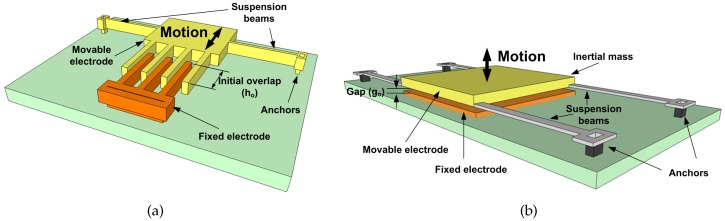
Schematic of (**a**) in-plane and (**b**) out-of-plane variable capacitors.

First, we compare the energy harvesting capabilities of the two most prevalent variable capacitor implementation: in-plane comb-finger, Figure 2a, and out-of-plane parallel-plates, Figure 2b. The capacitance of a comb-finger capacitor is:

(3) $$C_{v}=(1-\frac{x}{h_{\circ }})C_{\circ }$$

where $C_{\circ }$ is the capacitance at the initial finger length $h_{\circ }$ and x(t) is the movable electrode displacement. The capacitance of parallel-plate capacitors is:

(4) $$C_{v}=\frac{C_{\circ }}{1-x/g_{\circ }}$$

where $C_{\circ }$ is the capacitance at the initial gap $g_{\circ }$ between the electrodes and x(t) is the displacement of the movable electrode.

**Figure 3:**
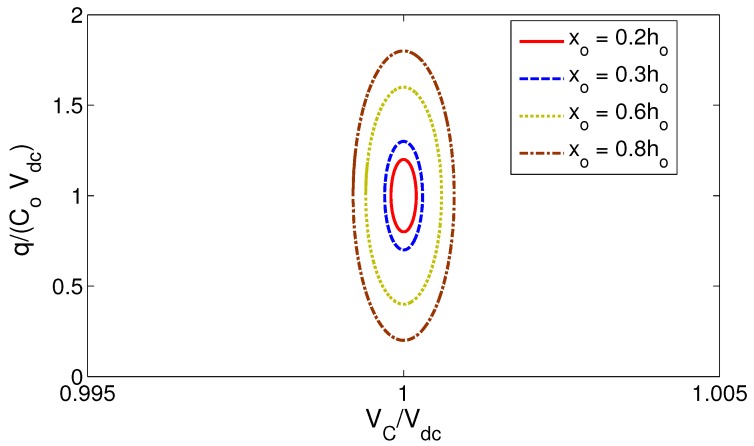
The q-V_C_ curves for the in-plane comb-finger transducer for various electrode displacement amplitudes.

**Figure 4:**
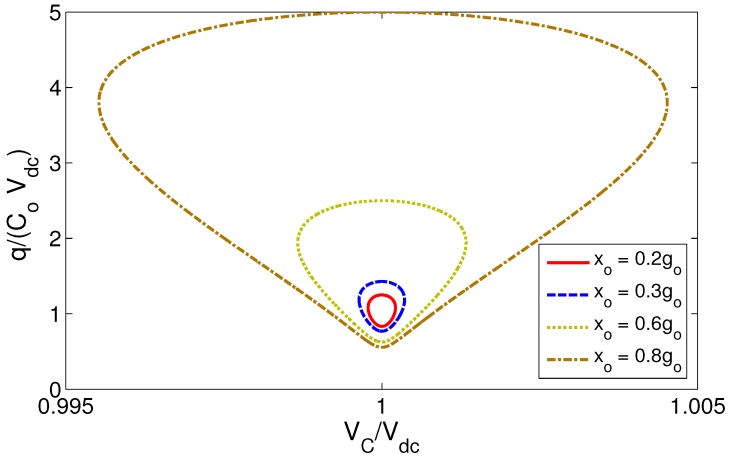
The q-V_C_ curves for the out-of-plane parallel-plate transducer for various electrode displacement amplitudes.

Assuming a sinusoidally-moving electrode:

(5) $$x\left(t\right)=x_{\circ }sin\left(\Omega t\right)$$

where $x_{\circ }$ is the peak displacement, we can evaluate the area enclosed by the variable capacitor charge-voltage (q-V_C_) curve to determine the amount of electrical energy harvested per conversion cycle. The curves are obtained by substituting Equation (5), Equation (3) for the in-plane comb-finger transducer and Equation (4) for the out-of-plane parallel-plate transducer into Equation (2) and integrating the resulting equation for a cycle. The q-V_C_ curves for the in-plane comb-finger and out-of-plane parallel-plate are shown in Figure 3 and Figure 4, respectively. Electrode displacements are normalized with respect to the maximum stroke $h_{\circ }$ and $g_{\circ }$, respectively; voltage is normalized with respect to $V_{dc}$; charge is normalized with respect to $C_{\circ }V_{dc}$; and therefore, the harvested energy is equal to the area enclosed by the loci times $C_{\circ }V_{dc}^{2}$; the magnitude of the initial (static) electric energy.

Comparing the two figures, it is clear that for the same normalized stroke (input vibrations), the singularity in out-of-plane parallel-plate transducers, as motion size becomes comparable to the gap $x_{\circ }\to g_{\circ }$, allows them to harvest much more electrical energy per conversion cycle than in-plane comb-finger transducers. We conclude that out-of-plane parallel-plate transducers are more effective in realizing continuous micro-power generators than conventional in-plane comb-finger transducers and adopt them for this work.

**Figure 5:**
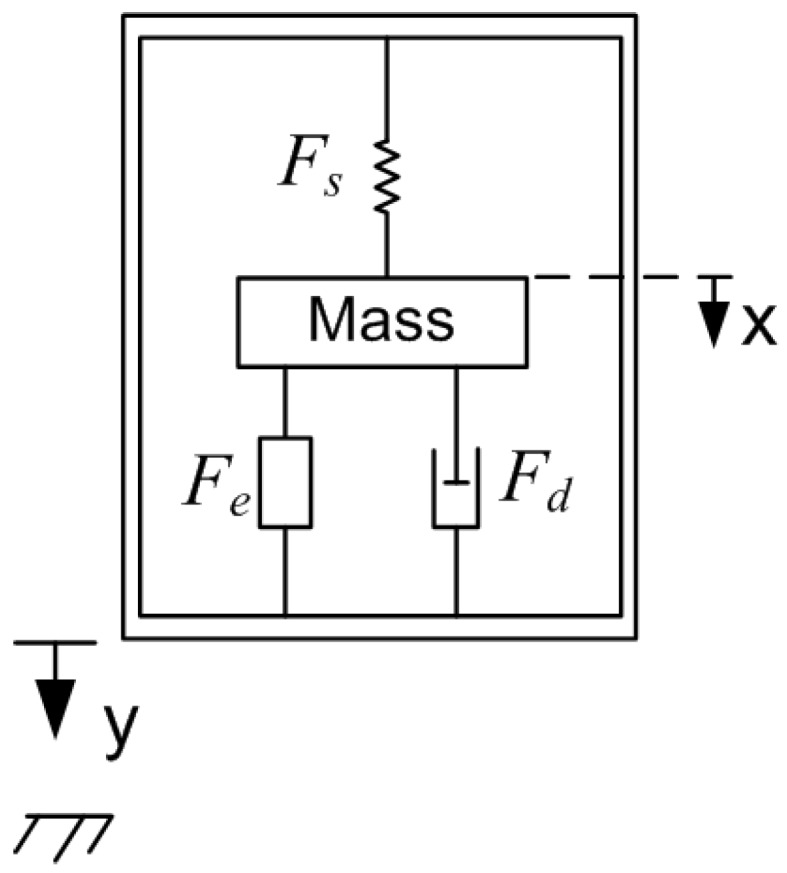
Schematic of a vibration energy harvester.

To study the full system dynamics, the electrical model of the transducer, Equation (2), is augmented with an electromechanical model to describe the capture of kinetic energy. Vibration energy harvesters, shown schematically in Figure 5, use an inertial mass *m* to capture base excitations y(t). In electrostatic harvesters, the inertial mass is attached to a movable electrode supported by a spring that exerts a force $F_{s}$. The electrostatic field exerts a force $F_{e}$ to attract the moving electrode to the fixed electrode while a damper opposes the motion, of the mass with a damping force $F_{d}$. Using Newton’s second law, the equation of motion of the inertial mass can be written as [20]:

(6) $$m\ddot{x}=\frac{q^{2}}{2g_{\circ }C_{\circ }}-k_{11}x-c_{m}\dot{x}-ma_{\circ }\sin\left(\Omega t\right)$$

where the capacitor plates are assumed rigid and parallel, the spring is assumed linear with a stiffness of $k_{11}$ and $a_{\circ }$ and Ω are the amplitude and frequency of base acceleration. The damping force is composed of linear viscous damping $c_{l}$ and nonlinear squeeze-film damping $c_{sq}$:

(7) $$c_{m}=c_{l}+c_{sq}$$

Squeeze-film damping depends on the gap between the electrodes. It can be represented by [19]:

(8) $$c_{sq}=\frac{3}{2\pi }\frac{\mu A^{2}}{{\left(g_{\circ }-x\right)}^{3}}$$

where μ and *A* are air viscosity and the electrodes’ surface area, respectively.

Substituting Equations (7) and (8) into Equation (6) and using Equation (4) in Equation (2), the system dynamics are described by:

(9) $$\begin{array}{ccc} \dot{q} & = & -\frac{q}{RC_{\circ }}(1-\frac{x}{g_{\circ }})+\frac{V_{dc}}{R}\\ \ddot{x} & = & \frac{q^{2}}{2mg_{\circ }C_{\circ }}-\omega_{m}^{2}x-(2\zeta_{l}\omega_{m}-\frac{3}{2\pi }\frac{\mu A^{2}}{m{\left(g_{\circ }+x\right)}^{3}})\dot{x}-a_{\circ }\sin\left(\Omega t\right) \end{array}$$

where $\omega_{m}=\sqrt{\frac{k_{11}}{m}}$ is the mechanical natural frequency and $\zeta_{l}=\frac{c_{l}}{2\sqrt{k_{11}m}}$ is the damping ratio.

This nonlinear system of differential equations represents a lumped-mass model of the energy harvester in free-flight. It does not account for the possibility of the inertial mass coming into contact with the fixed electrode, which is addressed later.

## 3. Model Validation

The prototype used to demonstrate the out-of-plane electrostatic energy harvester was fabricated using precision machining. It consists of a bottom fixed electrode and an upper movable electrode supporting a steel inertial mass $m_{1}$ with dimensions of: 9 mm × 2 mm × 1.8 mm. The movable electrode is attached to the anchors using four aluminium beams. The anchors are fixed to the base using screws. The gap between the electrodes is varied by inserting shims between the anchors and the base to obtain the corresponding gap. Table 1 lists the prototype dimensions.

The linear spring constant of guided beams can be estimated using the Equation [19]:

(10) $$k_{11}=nE\frac{bh^{3}}{L^{3}}$$

where *n*, *E*, *b*, *h* and *L* are the number, Young’s modulus, width, thickness and length of the beams, respectively. Using Equation (10) and the beam dimensions listed in Table 1, $k_{11}$ is calculated as 13,361 N/m. The natural frequency is calculated using the inertial mass $m_{1}$ from Table 1 as $\omega_{b}=673$ rad/s or $f_{b}=107$ Hz.

**Figure 6:**
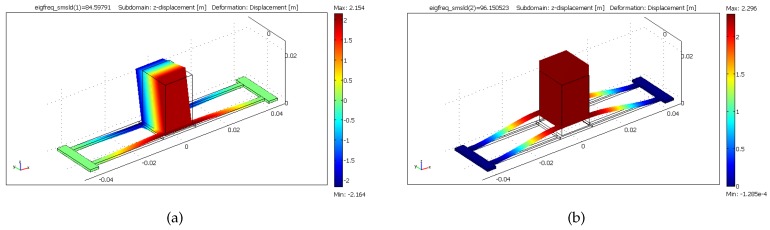
The (**a**) first and (**b**) second mode shapes of the prototype.

To account for the non-idealized geometry and configuration of the beams, nonlinear finite element analysis (FEA) is carried out on the prototype using COMSOL. The linear spring constant is then extracted from the relationship between the static force and displacement to be 10,987 N/m. The first and second mode shapes of the harvester prototype are also found using COMSOL and shown in Figure 6. The first mode is a torsional mode and occurs at $f_{t}=84.6$ Hz. The second mode is a bending mode and exists at $f_{b}=96.1$ Hz. The bending mode is used for energy harvesting; therefore, the effective mass of the energy harvester is calculated from the second mode natural frequency and the linear spring constant as m=30.1 gm.

**Figure 7:**
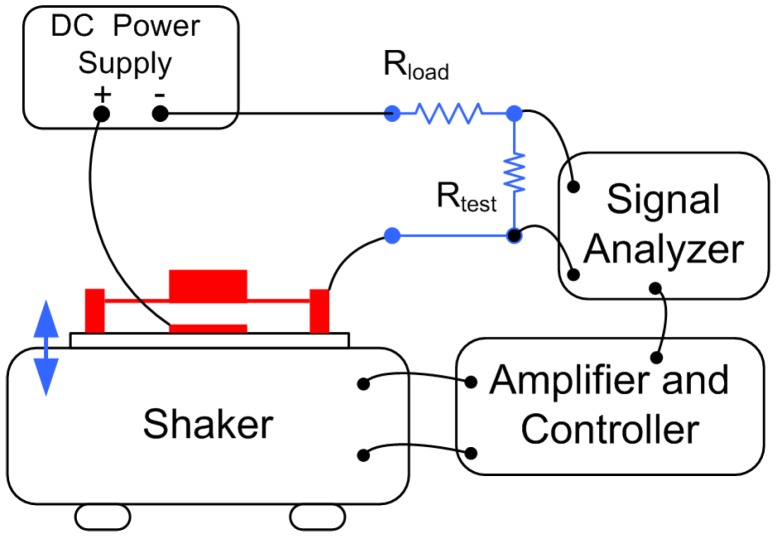
The experimental setup of the energy harvester prototype.

A schematic of the experimental setup is shown in Figure 7. The prototype is placed on the base of a pneumatic shaker, which is used to supply base excitations. The prototype is connected electrically to a DC power supply and load and test resistors. The test resistor $R_{test}$ is used in series with the load resistor $R_{load}$ to prevent the loading effect of the signal analyser input impedance. The signal analyser measures the root mean square (RMS) of the output voltage across the test resistor.

**Figure 8:**
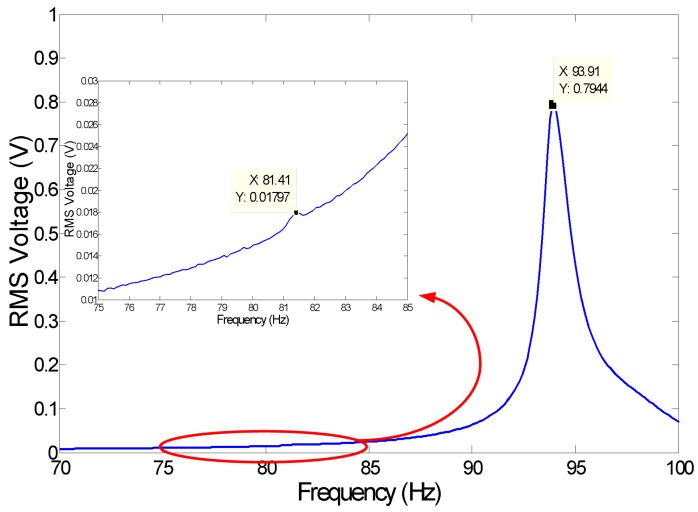
RMS of the output voltage obtained experimentally from a frequency sweep of base acceleration at an amplitude of 0.04 g (RMS).

The gap between the electrodes is initially set to a nominal value of 250 μm, the DC power supply to 300 V and load resistance of R=1.1 MΩ. The load resistance is then broken into primary load and test resistances 1 MΩ and 100 KΩ, respectively. The frequency-response of the RMS output voltage is obtained by sweeping the frequency of base accelerations between 70 Hz and 100 Hz while holding the amplitude constant at $a_{\circ }=0.04$ g (RMS). Two peaks are observed in the frequency-response curve, Figure 8, at $f_{t}=81.41$ Hz and $f_{b}=94.1$ Hz. The first peak corresponds to the torsional mode, while the second peak corresponds to the bending mode. The voltage level of the torsional mode is much smaller than that of the bending mode since torsional motions of the movable electrode do not produce as much variation in the capacitance as up-and-down motions of the bending mode.

The lower natural frequencies obtained experimentally are due to support flexibility (anchors, screws and base), unaccounted for in the FE model. Using the effective mass calculated from FEA and the natural frequency of the second mode obtained experimentally, the linear spring constant is calculated as $k_{11}=$ 10,480 N/m.

In order to compare the experimental results with the model, the actual gap $g_{\circ }$ and the damping coefficients need to be estimated. The actual gap differs from the nominal gap by the amount of static deflection that occurs because of settling under the weight of the inertial mass. The static deflection is evaluated as:

(11) $$x_{static}=\frac{mg}{k_{11}}$$

where *g* is the acceleration of gravity. The static deflection of the prototype is calculated as 25 μm; therefore, $g_{\circ }$ = 225 μm.

The total damping ratio can be extracted from the experimental results using the half power bandwidth BW and natural frequency $f_{\circ }$:

(12) $$\zeta_{m}=\frac{BW}{2f_{\circ }}$$

Using the results in Figure 8, it is found that $\zeta_{m}=0.006$. A parameter estimation procedure is developed to estimate $\zeta_{l}$ based on the fact that squeeze-film damping is minimal for electrode motions away from the fixed electrode. The procedure uses the frequency-sweep corresponding to the lowest excitation amplitude available in the dataset; in this case, corresponding to a base acceleration of $a_{\circ }$ = 0.02 g (RMS). The total damping coefficient $\zeta_{m}$ is then used as an initial guess for the linear damping coefficient $\zeta_{l}^{i}=\zeta_{m}$. The energy harvester model Equation (9) is integrated numerically for the output voltage at resonance, and the value of the linear damping coefficient is reduced until the values of the numerical and experimental RMS output voltage match.

**Figure 9:**
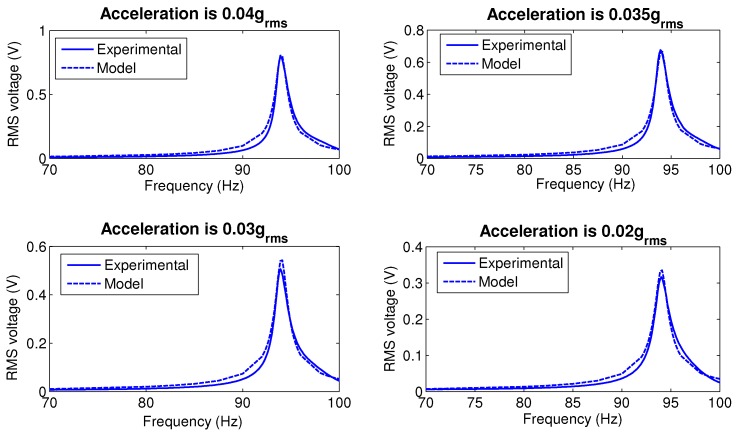
The frequency-response curves of the RMS output voltage at four base acceleration amplitudes obtained experimentally (solid lines) and numerically (dotted lines).

Using this procedure, we found the value of the linear damping coefficient to be $\zeta_{l}=0.0057$. Figure 9 compares the frequency-response curves of the RMS output voltage for base acceleration amplitudes of $a_{\circ }$ = 0.02 g, 0.03 g, 0.035g and 0.04 g (RMS). The curves shown in solid lines were obtained experimentally, while the curves shown in dotted lines were obtained by numerically integrating Equation (9) for the parameters estimated above. The results show good qualitative and quantitative agreement between the model and experiment for all four excitation levels.

**Figure 10:**
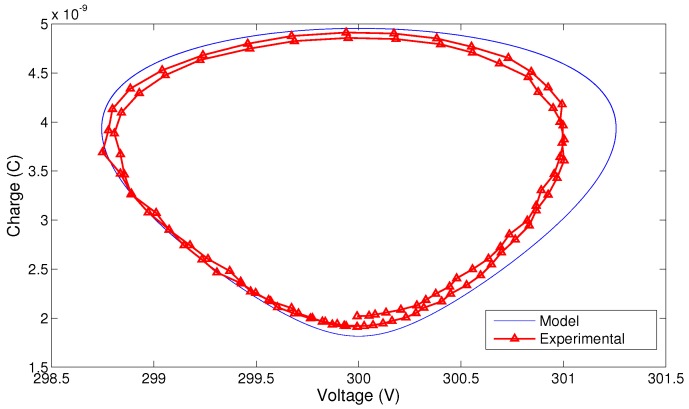
The q-V_C_ curves for the energy harvester prototype at $a_{\circ }$ = 0.04 g (RMS) and R=1.1 MΩ.

Finally, the q-V_C_ curve is found experimentally and predicted using the model (9) at the natural frequency of the bending mode $f_{b}=94.1$ Hz. The experimental voltage across the variable capacitor is obtained as the difference between the measured supply and load voltages. The charge on the variable capacitor is obtained by integrating the current measured passing through the load. The constant of integration is estimated by shifting the experimental q-V_C_ curve along the Q-axis to fit within the q-V_C_ curve obtained by numerical integration of the model Equation (9). Figure 10 shows the experimental and numerical q-V_C_ curves for a base acceleration amplitude of $a_{\circ }$ = 0.04 g (RMS). The areas enclosed by the two curves are close to each other. The experimental results are moved up along the Q-axis by adding a constant of integration $Q_{\circ }=0.72\;C_{\circ }V_{dc}$. This value correspond to the charge available on the variable capacitor at equilibrium (in the absence of motion). The fact that $Q_{\circ }<\;C_{\circ }V_{dc}$ is an indicator of the presence and relative magnitude of parasitic capacitance.

The experiment and model predications presented in this section show good agreement indicating the validity of the model developed in the previous section to describe continuous out-of-plane energy harvesters. In the next section, a practical implementation of an electrostatic micro-power generator (MPG) based on this energy harvester is introduced and studied.

## 4. MPG Realization

Realization of a practical electrostatic energy harvester requires the substitution of the DC voltage source with a portable charging source. A charged dielectric embedded within the structure of the transducer will be used to induce charges on the capacitor electrodes. A permanently-charged dielectric, an electret [21], constitutes an attractive option to satisfy this requirement since it facilitates sustainable operation over several years. Ready-made electrets are used here as a low cost solution to provide $V_{dc}$ that avoids extra fabricating and charging steps.

**Figure 11:**
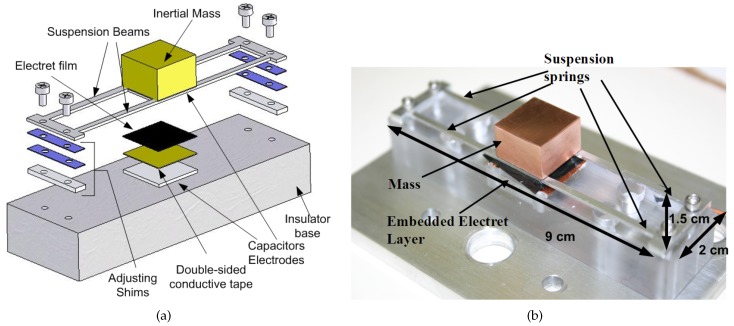
(**a**) Extended 3D schematic of the electret-MPG prototype and (**b**) out-of-plane variable capacitors.

An electret film is integrated into the energy harvester prototype introduced in Section 3 to constitute an electret-MPG prototype. Figure 11a shows an extended 3D schematic of the electret-MPG prototype where the electret layer is attached to the top surface of the fixed electrode using conductive double-sided copper tape. The electret layer is fashioned from a negatively-charged PTFE Teflon sheet (Rad Elec Inc., Frederick, MD , USA). It has a thickness of 50 μm, and and the surface voltage was measured at $V_{dc}$ = −410 V.

Figure 11b shows a picture of the assembled MPG prototype. The MPG prototype is identical to the energy harvester prototype except that the inertial mass is initially made of a shorter copper block with $m_{2}=17.5$ gm resulting in an effective mass of m=20.4 gm. The reduced rotary inertia of the new mass increases the natural frequency of the torsional mode, so that it exceeds the natural frequency of the bending mode. FE analysis calculates the natural frequency of the bending mode at $f_{b}=121$ Hz and the natural frequency of the torsional mode at $f_{t}=170$ Hz. The test setup of the MPG prototype is identical to that of the energy harvester shown in Figure 7, except that the external DC power source is replaced with an electret film.

**Figure 12:**
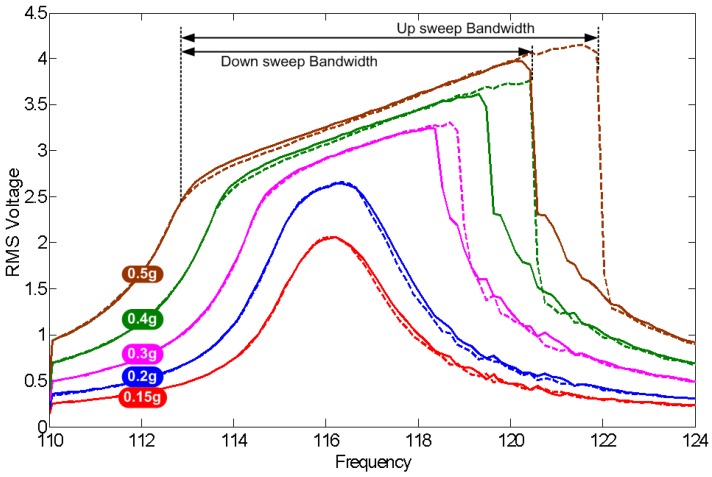
Frequency response of the RMS output voltage for inertial mass $m_{2}$, $g_{\circ 1}$ = 300 μm, and base acceleration amplitudes in the range of $a_{\circ }$ = 0.15‒0.5 g (RMS). Frequency up-sweeps are shown in dashed lines and down-sweeps in solid lines.

The nominal capacitor gap is initially set equal to $g_{\circ 1}$ = 300 μm by inserting shims under the anchors. The frequency response of the system is obtained by sweeping the frequency of base acceleration while maintaining the amplitude constant. The frequency is swept up and down over the whole frequency range of [110,124] Hz to detect the presence of hysteresis in the system response. Figure 12 shows the frequency response of the RMS output voltage for different vibration base acceleration amplitudes of $a_{\circ }$ = 0.15 g, 0.2 g, 0.3 g, 0.4 g and 0.5 g (RMS).

The frequency-response curves in Figure 12 show three distinct regions of behaviour: linear, nonlinear and impact. Linear response is seen at low acceleration amplitudes ($a_{\circ }$ = 0.15 g) and is characterized by a frequency-response curve centred around the natural frequency of the bending mode $f_{b}=116$ Hz. Similar to the case of the energy harvester, the natural frequency obtained experimentally is lower than that obtained by FEA because of unmodeled support compliance.

Nonlinear response is seen at acceleration amplitude of $a_{\circ }$ = 0.2 g where the peak of the frequency-response curve appears wider than that of linear response due to the dominance of a nonlinear damping mechanism, squeeze-film, for large motions (near resonance) as the moving plate approaches the electret layer. Impact response is seen for base acceleration amplitudes $a_{\circ }$≥ 0.25 g. The onset of impact is marked by a ‘knee’, an abrupt change in the curvature of the frequency-response curve.

Defining the MPG bandwidth as the half-power bandwidth for linear response and the distance between the response knees for nonlinear and impact responses, we observe the following:

- The MPG bandwidth increases as the amplitude of base acceleration increases, and the response region changes from linear to nonlinear to impact;
- The up-sweep and down-sweep bandwidth are equal for linear and nonlinear responses while the up-sweep bandwidth is wider than the down-sweep bandwidth for impact response, in agreement with Soliman et al. [22].

Specifically, Figure 12 shows that the MPG bandwidth increases from 2 Hz under linear response to 9 Hz under impact and a base acceleration amplitude of $a_{\circ }$ = 0.5 g. These results suggest the use of impacting electrostatic MPGs as wideband MPGs [23] that can harvest more energy by increasing the harvesting bandwidth and, therefore, the fraction of time during which environmental vibrations are harvested.

**Figure 13:**
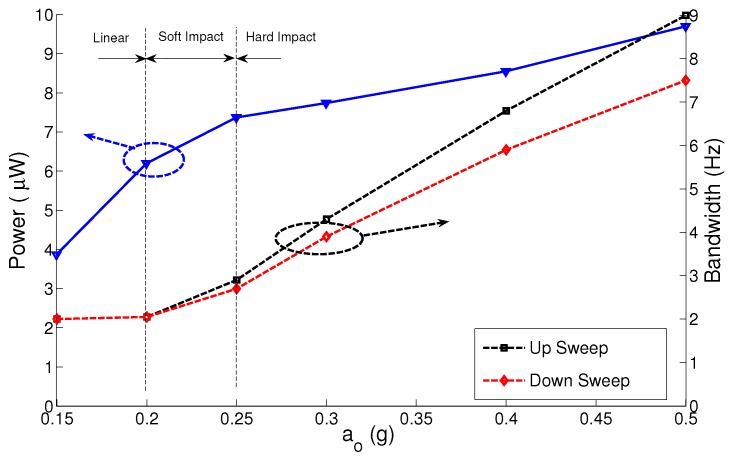
Output power and bandwidth of the micro-power generator (MPG) prototype as a function of base acceleration amplitude at $g_{\circ 1}$ = 300 μm.

**Figure 14:**
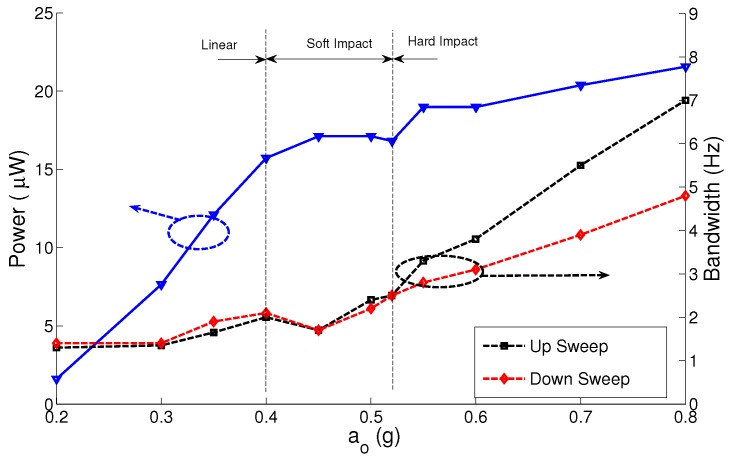
Output power and bandwidth of the MPG prototype as a function of base acceleration amplitude at $g_{\circ 2}$ = 425 μm.

**Figure 15:**
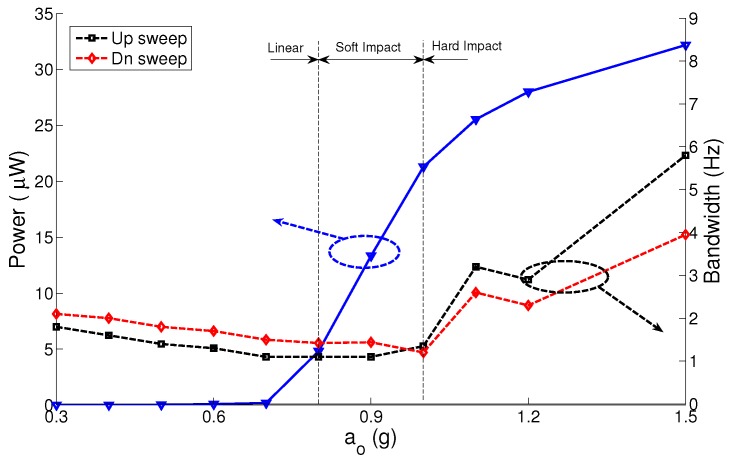
Output power and bandwidth of the MPG prototype as a function of base acceleration amplitude at $g_{\circ 3}=750$ μm.

To test the effect of variation in the electrostatic field strength on the MPG performance, we repeat the experiment shown in Figure 12 using the MPG prototype with nominal capacitor gaps of $g_{\circ 2}=425\;\mu$m and $g_{\circ 3}=750\;\mu$m. The results for the three gap distances are summarized in Figure 13, Figure 14 and Figure 15. The figures show the MPG output power at the natural frequency of the prototype $f_{b}=116$ Hz and bandwidth in frequency up- and down-sweeps at a constant base acceleration amplitude.

The results show that the MPG output power and bandwidth depend on the interaction between three factors: the strength of the electrostatic field, squeeze-film damping and impact. A small gap and, thus, a stronger electrostatic field for a given electret voltage allow the MPG to extract more output power from lower excitation levels than MPGs with weaker electrostatic fields. This can be seen by comparing the output power of the MPG at $g_{\circ 1}$ to those at $g_{\circ 2}$ and $g_{\circ 3}$ at low base acceleration amplitudes $a_{\circ }$ ≤ 0.2 g. The output power is more than 6 μW for $g_{\circ 1}$ and less than 10 nW for $g_{\circ 3}$ at base acceleration amplitude of $a_{\circ }$ = 0.2 g. That is because the output power is proportional to the nominal capacitance $C_{\circ }$. Similarly a smaller gap increases squeeze-film damping, even in the linear region, which increases the mechanical losses in the MPG. This can be seen in the larger bandwidth of the linear region for $g_{\circ 1}$, BW = 2 Hz, than those for $g_{\circ 2}$ and $g_{\circ 3}$.

A smaller gap leads to low impact losses since it means that impact will occur at a lower velocity. This can be seen in the figures by comparing the up-sweep bandwidth for $g_{\circ 1}$, BW = 9 Hz at $a_{\circ }$ = 0.5 g, to that for $g_{\circ 3}$, BW = 5.75 Hz at $a_{\circ }$ = 1.5 g. Soliman et al. [23] show that the up-sweep bandwidth is counter-proportional to impact losses. On the other hand, a larger gap allows for a longer stroke and a higher peak velocity resulting in higher output power. This can be seen in the value at which the output power saturates with increasing base acceleration amplitude for the three gap distances. The output power saturates at 8 μW, 20 μW and 30 μW for $g_{\circ 1}$, $g_{\circ 2}$ and $g_{\circ 3}$, respectively.

We conclude that it is crucial to balance these three factors in the design of out-of-plane electrostatic MPGs. The proper balance depends on the harvesting environment

- For environments where small amplitude vibrations are available, a small capacitor gap should be used to increase the strength of the electrostatic field and obtain higher nominal capacitance $C_{\circ }$ while maintaining the response in the linear region, thereby avoiding impact loses and increasing the efficiency of power extraction from low excitation amplitudes.
- For environments where large amplitude vibrations are available in a narrow frequency band, a large capacitor gap combined with a strong electrostatic field generated by a large source voltage will lead to high efficiency energy extraction by maintaining the response near the linear response region while guaranteeing a larger output power saturation level by allowing for a larger stroke.
- For environments where large amplitude vibrations are available in a wide frequency band, a large capacitor gap will lead to larger output power and wider MPG bandwidth by operating in the impact region.

## 5. Impact Model

Operating the MPG in the impact region creates a wideband MPG with up to 9 Hz in the up-sweep and 7 Hz in the down-sweep bandwidth. Therefore, it is important to develop an MPG model valid for the impact region for use in performance prediction and optimization. In this section, a modified system model is developed and verified by comparison to experimental results.

Manual assembly of the electret on the bottom electrode creates bumps on the electret surface. To account for this, the electret film position is elevated, thereby reducing the effective gap to $g_{i}$. Further, two linear viscous damping coefficients are defined to capture the dissipative processes during the flight $c_{m}$ and impact $c_{i}$ phases of motion:

(13) $$F_{d}=\left\{\begin{array}{cc} c_{m}\dot{x} & x<g_{i}\\ c_{i}\dot{x} & x\geq g_{i} \end{array}\right.$$

The restoring force $F_{s}$ is also re-defined to encompass the impact phase of motion as follows:

(14) $$F_{s}=\left\{\begin{array}{cc} k_{11}x+k_{13}x^{3} & x<g_{i}\\ k_{2}x+\left(k_{11}-k_{2}\right)g_{i}+k_{13}{g_{i}}^{3} & x\geq g_{i} \end{array}\right.$$

where $k_{11}$ and $k_{13}$ are the linear and cubic stiffness coefficients of the suspension beams and $k_{2}$ is the stiffness coefficient of impact with the electret-covered bottom electrode.

Using Equations (13) and (14), a model is obtained for the MPG in the impact region:

(15) $$\begin{array}{ccc} \dot{q} & = & -\frac{q}{RC_{\circ }}\left(1-\frac{x}{g_{\circ }}\right)+\frac{V_{dc}}{R} \end{array}$$

(16) $$\begin{array}{ccc} m\ddot{x} & = & \left\{\begin{array}{cc} \frac{1}{2}\frac{q^{2}}{C_{\circ }g_{\circ }}-k_{11}x+k_{13}x^{3}-c_{m}\dot{x}-m\ddot{y} & x<g_{i}\\ \frac{1}{2}\frac{q^{2}}{C_{\circ }g_{\circ }}-k_{2}x-\left(k_{11}-k_{2}\right)g_{i}-k_{13}{g_{i}}^{3}-c_{i}\dot{x}-m\ddot{y} & x\geq g_{i} \end{array}\right. \end{array}$$

Realization of the impact model requires estimation of the system parameters. The linear $k_{11}$ and cubic $k_{13}$ stiffness coefficients are extracted by fitting a third-order polynomial to the static force-deflection curve obtained from nonlinear FEA of the MPG with inertial mass $m_{2}$. The linear stiffness coefficient is then used in conjunction with the natural frequency of the bending mode obtained from FEA ($f_{b}=121$ Hz) to extract the effecting mass of the MPG (m=19.5 gm). The linear stiffness coefficient was then reduced to match the natural frequency of the bending mode to the experimentally-measured value, $f_{b}=116$ Hz, thereby accounting for the compliance in the supports. The damping coefficient of the free flight phase $c_{m}$ is calculated from $\zeta_{m}$ obtained from the experiment using Equation (12) and the definition of the damping ratio:

(17) $$c_{m}=2\zeta_{m}\sqrt{k_{11}m}$$

The effective gap $g_{\circ }$ is obtained by matching the RMS output voltage of the model to the same experiment at a frequency away from resonance (f=110 Hz).

The linear stiffness of the contact spring $k_{2}$ is found by matching the slope of the experimental and numerical frequency-response curves of the output voltage during impact at a base acceleration of $a_{\circ }=0.5$ g. The damping coefficient during impact $c_{i}$ is found by matching the response of the up-sweep during the same experiment. The impact height $g_{i}$ is estimated by matching the left knee in the frequency-response curve predicted by the model to that obtained from the experiment. The estimated model parameters are listed in Table 2.

**Figure 16:**
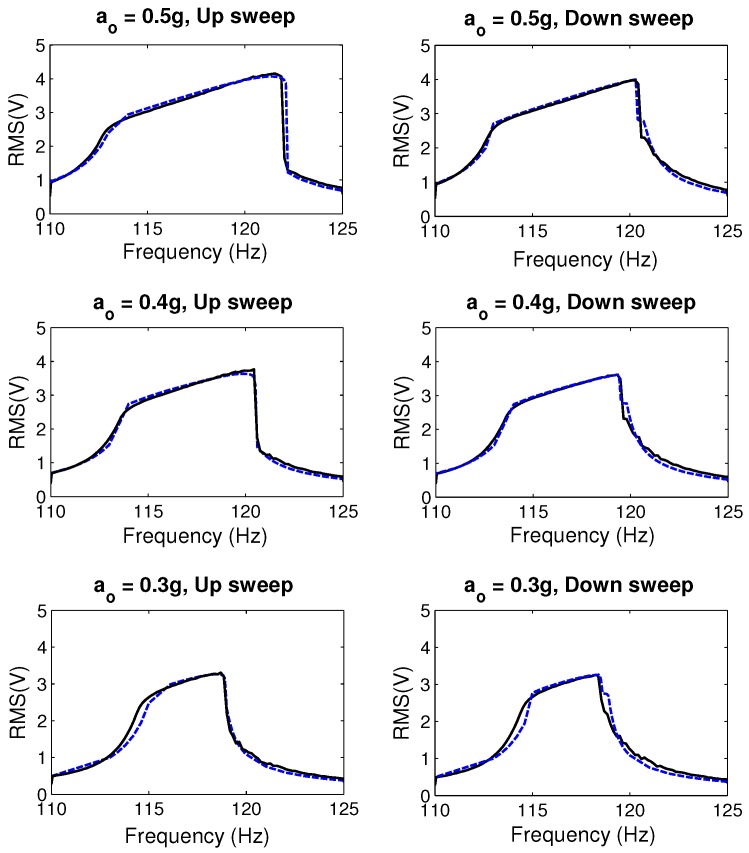
Experimental (solid lines) and impact model predicted (dashed lines) frequency-response curves of the RMS output voltage for the MPG prototype with inertial mass $m_{2}$ and gap $g_{\circ 1}$.

Figure 16 compares the frequency-response curves obtained from the impact model and the experiment in frequency up- and down-sweeps of the MPG prototype with inertial mass $m_{2}$ and gap $g_{\circ 1}$. The figures show good agreement between model predictions and experimental results.

## 6. Improved MPG

We examine the potential to design better MPGs that can capture more kinetic energy from the environment by testing the effect of stronger electrostatic fields and larger inertial mass. The strength of the electrostatic field is increased using a high-voltage electret at a similar gap to the previous experiment to minimize the effects of variation in squeeze-film damping and impact losses on the MPG performance. The surface voltage of the charged electret film was measured upon receipt from the manufacturer at −1700 V. At the time of the previous experiment, the surface voltage had degraded and was measured at −410 V. The surface voltage is known to drop in open air due to charge recombination under the influence of humidity [21]. For this experiment, we use an electret that was sealed from air until use in the experiment to preserve the charge within the electret. We also use a tall inertial mass $m_{1}=29.5$ gm and a short inertial mass $m_{2}=17.5$ gm. The nominal gap after installing the fresh electret is $g_{o4}=275\;\mu$m.

**Figure 17:**
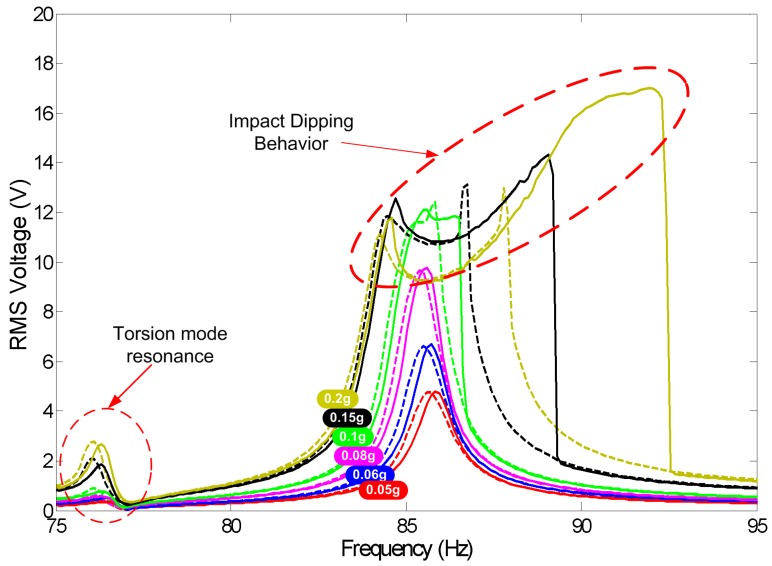
Frequency response of the RMS output voltage for inertial mass $m_{1}=29.5$ gm and base acceleration amplitudes in the range $a_{\circ }=0.05$‒0.2 g (RMS). Frequency up-sweeps are shown in solid lines and down-sweeps in dashed lines.

Figure 17 shows the frequency response of the RMS output voltage using the fresh electret, the tall inertial mass $m_{1}$ and base acceleration amplitudes in the range $a_{\circ }=0.05$‒0.2 g (RMS). Comparing Figure 17 to Figure 12, it can be seen that higher source voltage and larger inertial mass increase the output voltage by an order-of-magnitude for the same input base acceleration. It also results in the impact region starting at lower excitation levels (≤0.1 g) producing a wider MPG bandwidth even for low environmental vibration amplitudes. We note that the higher rotary inertia of the tall mass $m_{1}$ shifts the natural frequency of the torsional mode $f_{t}=76$ Hz below the natural frequency of the bending mode $f_{b}=86$ Hz. This configuration has a detrimental effect on the MPG performance in the impact region. This can be seen in the initial drop in the output voltage beyond the left “knee” of the frequency response curve instead of the the gradual increase observed in Figure 12. In this initial region, impact couples the closely-spaced bending and torsional modes channelling some of the kinetic energy to the torsional mode, which is less effective in energy harvesting than the bending mode. In fact, the time-history of the output voltage shows a signal at the forcing frequency Ω modulated by the torsional mode natural frequency $f_{t}$.

**Figure 18:**
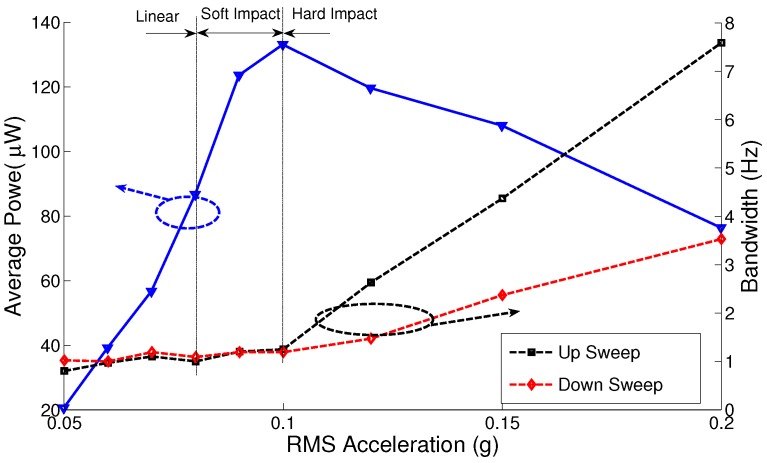
MPG prototype’s average power and bandwidth at different accelerations for $m_{1}$.

Figure 18 shows the output power and MPG bandwidth at the natural frequency of the bending mode $f_{b}=86$ Hz for the fresh electret, inertial mass $m_{1}$ and base acceleration amplitudes in the range $a_{\circ }\;=0.05$‒0.2 g (RMS). The bandwidth of the MPG increases linearly with the amplitude of base acceleration in the impact region. On the other hand, the output power drops progressively in the impact region as higher excitation amplitudes channel more kinetic energy into the inefficient bending mode.

**Figure 19:**
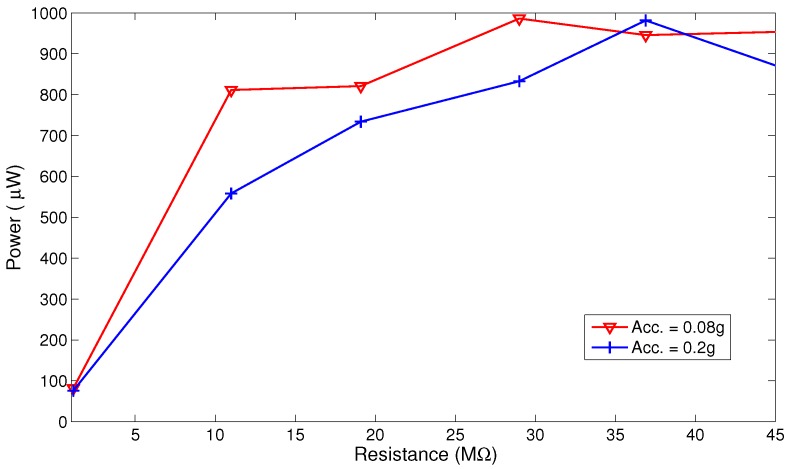
Average output power as a function of the load resistance *R* for inertial mass $m_{1}$ and $g_{o4}$.

**Figure 20:**
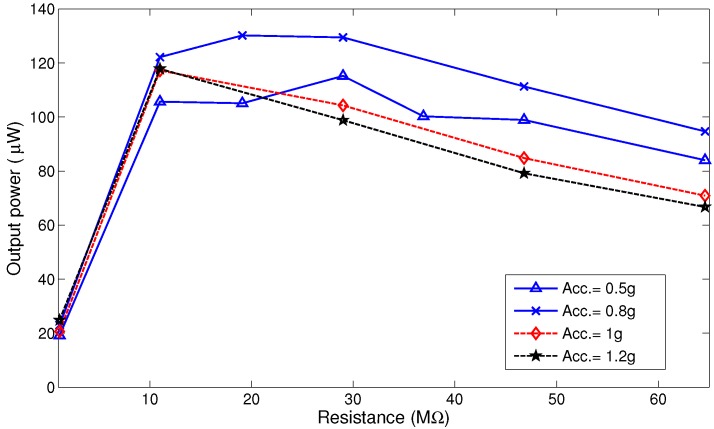
Average output power as a function of the load resistance *R* for inertial mass $m_{2}$ and $g_{o2}$ (solid lines) and $g_{o3}$ (dashed lines).

We note that while increasing the inertial mass allows the MPG to capture more kinetics, it changes the optimal load resistance as shown in Figure 19 and Figure 20. Figure 19 shows that changing the load resistance from the nominal value R=1.1 MΩ increases the output power from less than 100 μW to more than 900 μW in the range R=30‒37 MΩ. Figure 20 shows that decreasing the inertial mass to $m_{2}$ decreases the maximum output power to 130μW and the optimal load resistance to the range R=10‒20 MΩ.

## 7. Conclusions

The results and analysis presented above demonstrate the feasibility and advantages of electret-based out-of-plane continuous MPGs. In fact, the improved MPG proves this potential by realizing almost 1 mW of output power in Figure 19 at $a_{\circ }=0.08$ g (RMS) base acceleration amplitude. In addition, the implementation and fabrication of this PG are simple and use low-cost components. Table 3 compares the performance of this electrostatic MPG to previously reported electrostatic MPGs. The results show that the improved prototype generates a power density closer to [24], but at a much lower center frequency $f_{\circ }$ and excitation level. Further, several engineering enhancements can be easily introduced to minimize the volume of the device and increase the power density. For example, using 0.2 mm instead of 0.9 mm-thick sheets to make the moving structure will cut the beams length by more than one third without changing the MPG center frequency and, thus, more than doubling the power density.

We find that the capacitor gap should be set to match the requirements of the harvesting environment. On the other hand, increasing the electrostatic field strength by using a high DC voltage source is always advantageous leading to more sensitive MPGs that can collect energy at lower excitation levels, larger optimum output power and wider MPG bandwidth. Larger nominal capacitance, and thus capacitor area, and inertial mass allow for a larger optimum output power; however, they change the nature of the electromechanical coupling in the MPG and require a search for the optimal load resistance at a particular configuration.

Finally, the use of an electret layer as a DC charging source does not only make the MPG portable and low cost, but also helps to isolate the two capacitive electrodes during impact. However, since negatively-charged electrets degrade with exposure to humidity, the implementation of a good sealing is a must to preserve the electret charge over the lifetime of the MPG.

## Figures and Tables

**Table 1:** The electrostatic harvester prototype dimensions.

Parameter	Value	Parameter	Value
Plate length	15 mm	Plate width	15 mm
Beam length	32 mm	Beam width	2 mm
Beam thickness	0.9 mm	Inertial mass $m_{1}$	29.5 gm

**Table 2:** Summary of the MPG impact model parameters.

Parameter	Value	Parameter	Value
$k_{11}$	10,443 N/m	$k_{13}$	7.1 × $10^{9}$ N/m
$k_{2}$	$8k_{11}$	$C_{\circ }$	8.1 pF
$c_{m}$	0.337 kg/s	$c_{i}$	78 $c_{m}$
$g_{i}$	240 μm	$g_{\circ }$	295 μm

**Table 3:** Comparison with previously published work.

ES MPG	Source	Size	Acceleration	*f*_∘_	Power Density
		(cm^3^)	(g)	(Hz)	(μW/cm^3^)
Bartsch et al. [12]	External	20.3	N/A	90	0.36
Edamoto et al. [8]	Electret	1.22	N/A	21	10.23
Hoffmann et al. [14]	External	0.2	13	1330	17.5
Kloub et al. [24]	External	0.17 ^1^	1	1740	29.8
This work	Electret	36	0.08	86	27.8

${}^{1}$ Packaging thickness is assumed to be 0.4 cm.
